# A degradatory fate for CCR4 suggests a primary role in Th2 inflammation

**DOI:** 10.1002/JLB.2A0120-089RR

**Published:** 2020-02-13

**Authors:** Caroline A. Anderson, Pallavi Patel, Jonathan M. Viney, Rhian M. Phillips, Roberto Solari, James E. Pease

**Affiliations:** ^1^ National Heart & Lung Institute Inflammation, Repair & Development Section Imperial College London London UK; ^2^ National Heart and Lung Institute Airway Disease Infection Section Imperial College London London UK; ^3^ Asthma UK Centre in Allergic Mechanisms of Asthma London UK

**Keywords:** chemokine receptor, chemokine, chemotaxis, endocytosis, Th2 lymphocyte

## Abstract

CCR4 is the sole receptor for the chemokines CCL22 and CCL17. Clinical studies of asthmatic airways have shown levels of both ligands and CCR4^+^ Th2 cells to be elevated, suggestive of a role in disease. Consequently, CCR4 has aroused much interest as a potential therapeutic target and an understanding of how its cell surface expression is regulated is highly desirable. To this end, receptor expression, receptor endocytosis, and chemotaxis were assessed using transfectants expressing CCR4, CCR4^+^ human T cell lines, and human Th2 cells polarized in vitro. CCL17 and CCL22 drove rapid endocytosis of CCR4 in a dose‐dependent manner. Replenishment at the cell surface was slow and sensitive to cycloheximide, suggestive of de novo synthesis of CCR4. Constitutive CCR4 endocytosis was also observed, with the internalized CCR4 found to be significantly degraded over a 6‐h incubation. Truncation of the CCR4 C‐terminus by 40 amino acids had no effect on cell surface expression, but resulted in significant impairment of ligand‐induced endocytosis. Consequently, migration to both CCL17 and CCL22 was significantly enhanced. In contrast, truncation of CCR4 did not impair constitutive endocytosis or degradation, suggesting the use of alternative receptor motifs in these processes. We conclude that CCR4 cell surface levels are tightly regulated, with a degradative fate for endocytosed receptor. We postulate that this strict control is desirable, given that Th2 cells recruited by CCR4 can induce the further expression of CCR4 ligands in a positive feedback loop, thereby enhancing allergic inflammation.

## Graphical abstract

Both ligand‐induced and constitutive endocytosis of CCR4 leads to intracellular proteolysis, with differing requirements for the CCR4 C‐terminus.

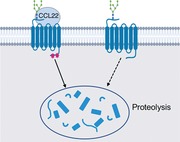

**Abbreviations**
ATLL: adult T‐cell leukemia/lymphoma
Benzyl GalNAc: benzyl *N*‐acetyl‐alpha‐d‐galactosaminide
CD: cluster of differentiation
CHO: Chinese hamster ovary
CHX: cycloheximide
ECL2: extracellular loop 2
GAPDH: glyceraldehyde 3‐phosphate dehydrogenase
GPCR: G protein‐coupled receptor
GRK: G protein‐coupled receptor kinase
HRP: horseradish peroxidase
MDC: Mϕ‐derived chemokine
TARC: Thymus and activation‐regulated chemokine
Treg: T regulatory cell
Tun: tunicamycin
WHIM: warts, hypergammaglubulinemia, infections, myelokathexis
WT: Wildtype
α‐CGRP: α calcitonin gene‐related peptide

## 1 INTRODUCTION

The directional movement of leukocytes during both homeostasis and disease is achieved through the actions of a family of small, secreted proteins known as chemokines, and their cognate G protein‐coupled receptors (GPCRs).^1^ Impaired or overactive chemokine signaling and subsequent leukocyte recruitment is associated with the pathogenesis of inflammatory diseases, infections, cancer, and autoimmunity.^2^ To prevent such dysregulated signaling a combination of processes, including receptor desensitization and endocytosis, receptor recycling, and/or receptor degradation, acts to modulate receptor signaling in response to changing chemokine concentrations in the surrounding microenvironment.^3^ The mechanisms regulating cell surface expression and function vary significantly among the chemokine receptor family, and may be indicative of the specific roles of individual receptors in health and disease.

Asthma is a chronic condition of the airways that causes significant morbidity and mortality worldwide, despite the availability of effective symptom‐relieving treatments.^4^ CCR4 has been widely implicated in the pathogenesis of inflammatory diseases such as asthma and atopic dermatitis due to its expression on Th2 cells.^5^, ^6^, ^7^ Following allergen provocation of the airways, the endogenous chemokine ligands of CCR4, CCL17/Thymus and activation regulated chemokine (TARC), and CCL22/Mϕ‐derived chemokine (MDC), are secreted by activated APCs, including dendritic cells, monocytes, and Mϕs.^8^ Gradients of these 2 chemokines lead to the recruitment of CCR4‐expressing, CD4^+^ Th2 cells, which infiltrate the lung as part of the late allergic response. Once there, Th2 cells contribute to the development of inflammation through the production of IL‐4, IL‐5, and IL‐13.^9^

CCL17 and CCL22 purportedly arose from a gene duplication event, yet share only 32% amino acid homology.^10^, ^11^, ^12^ Several studies have reported differences in the ability of these 2 chemokines to activate CCR4, providing evidence that they act as biased agonists of this receptor. Mariani et al initially reported that CCL22 but not CCL17 was able to induce CCR4 internalization in Th2 cells and CCR4 transfected cells.^13^ Conversely, Ajram et al reported that CCL22 but not CCL17 coupled CCR4 to β‐arrestin2 recruitment in a transfectant system.^14^ Studies of bronchial epithelial cells that also express CCR4 found that CCL17 induces significant transcription of the vasodilatory protein α‐calcitonin gene‐related peptide (CGRP) while CCL22 is ineffective.^15^ α‐CGRP is implicated in asthma pathogenesis,^16^ which is significant given that both CCL17 and CCL22 are increased in the bronchoalveolar lavage of asthmatics compared to healthy subjects.^7^

In this study, we examined the regulation of CCR4 expression, elucidating structural features of CCR4 that are important for this process. We found that CCR4 expression is controlled at multiple levels, suggestive of an important role for this receptor in tissue homeostasis and the control of Th2 inflammation, underscoring the potential for CCR4 targeting in the treatment of allergic disease.

## 2 METHODS

Unless otherwise stated, reagents were purchased from Thermo Fisher Scientific (Paisley, UK) or Sigma–Aldrich (Poole, UK). Recombinant human CCL17 and CCL22 were purchased from PeproTech EC (London, UK). The anti‐CCR4 Ab 10E4 was generated by Millennium Pharmaceuticals and has been previously described.^17^ The goat anti‐mouse FITC‐labeled F (ab’) 2 secondary Ab was purchased from Dako Cytomation (Cambridge, UK). The rabbit anti‐glyceraldehyde 3‐phosphate dehydrogenase (GAPDH) Ab was purchased from Millipore (MA, USA). pcDNA3.1 plasmids encoding either a wild‐type (WT) CCR4 construct or a C‐terminal truncation mutant of CCR4, CCR4‐Δ40, were generated by site‐directed mutagenesis as previously described.^18^

### 2.1 Cell lines

CHO‐K1 cells stably expressing CCR4 (CHO‐CCR4) have been previously described^14^ and were grown to confluency in DMEM Ham's F12 media supplemented with 10% FCS, 100 U/ml penicillin and 50 ng/ml streptomycin. Selection of CCR4‐expressing cells was maintained by addition of 1 mg/ml Geneticin. The L1.2 murine pre‐B lymphoma cell line and Hut78 T cell lymphoma cell line were maintained in liquid suspension in RPMI 1640 media supplemented with 10% heat‐inactivated FCS, 100 U/ml penicillin, 50 ng/ml streptomycin, 1 mM sodium pyruvate, 50 µM β‐mercaptoethanol, and non‐essential amino acids (RPMI Complete Media). To ensure transfection efficiency cells were maintained at a density of less than 1.0 × 10^6^ cells/ml. Simple media refers to RPMI media (for L1.2 and Hut78 cells), DMEM Ham's F12 media (CHO‐CCR4) and X‐VIVO 15 (Th2 cells) without the addition of FCS or sodium pyruvate, non‐essential amino acids, β‐mercaptoethanol, l‐glutamine, penicillin, or streptomycin.

### 2.2 Human Th2 cells

PBMCs were isolated from the whole blood of healthy donors, as previously described.^19^ Donors had provided informed consent in accordance with a protocol approved by the local ethics committee. CD4^+^ T cells were enriched from the isolated PBMC fraction by negative selection using an EasySep™ Human Naïve CD4^+^ T Cell Enrichment Kit (StemCell Technologies, Grenoble, France). To generate polarized Th2 cells, naïve CD4^+^ T cells were seeded onto 24‐well plates coated with anti‐human CD3 and cultured for 13 days in vitro in Th2 differentiation media, as per the instructions of the CellXVivo™ Human Th2 cell differentiation kit (Bio‐Techne, Oxford, UK).

### 2.3 Transient transfection of cells

L1.2 cells were transiently transfected with 1 µg plasmid DNA by electroporation, as previously described.^18^ Prior to assessing receptor expression, cells were cultured overnight in RPMI complete media with the addition of sodium butyrate to a final concentration of 10 mM, 5 h after electroporation. CHO‐CCR4 cells were transiently transfected with 2 µg plasmid DNA using Turbofect transfection reagents. Cells were harvested 24 h post‐transfection and transgene expression was assessed by flow cytometry.

### 2.4 Western blotting

Constitutive CCR4 degradation over the course of 6 h was assessed by incubating CHO‐CCR4 cells in serum‐free DMEM media containing 10 µg/ml cycloheximide at 37°C. The effects of the CCR4 ligands on CCR4 degradation were assessed by pre‐incubating CHO‐CCR4 cells for 30 min with 10 µg/ml cycloheximide prior to the addition of either CCL17 (100 nM) or CCL22 (100 nM) for 5 h at 37°C. Where inhibitors were used, cells were pre‐incubated with 10 µg/ml cycloheximide with the addition of either 43 or 1 µM Benzyl‐GalNac, DMSO vehicle control or 10 or 1 µg/mL tunicamycin for 30 min at 37°C. Cell lysates were generated and separated on 4–12% Bis‐Tris Protein gels as described previously.^20^ Gel transfer was performed using the iBlot dry transfer system. Non‐specific antigen binding was blocked using 5% milk powder in 1× TBST buffer for 1 h at room temperature. Blots were incubated overnight with 10E4 (diluted 1:2000 in blocking buffer) at 4°C. Following washing steps in 1× TBST cells were incubated with recombinant protein G‐HRP (diluted 1:4000 in 1× TBST) for 1 h at room temperature. Membranes were developed by chemiluminescence using ECL Plus substrate and imaged using a myECL imager. To obtain a loading control, membranes were stripped and re‐probed with GAPDH primary Ab overnight in blocking buffer.

In experiments comparing the constitutive degradation of WT CCR4 and CCR4‐Δ40 constructs it was observed that staining of the loading control GAPDH also degraded markedly over time in the presence of cycloheximide. In these experiments, the intensity of CCR4 staining was therefore normalized to the 0‐hour time‐point for each construct rather than to the GAPDH loading controls, which nonetheless demonstrated consistent protein loading for the first 3 h of the time‐course.

### 2.5 Flow cytometry

Cell surface CCR4 expression was measured using a FACS Calibur flow cytometer (BD), as previously described using the 10E4 anti‐CCR4 Ab.^21^

### 2.6 Internalization assay

For the assessment of the rate of constitutive CCR4 internalization, CHO‐CCR4 and Hut78 cells were pre‐incubated in serum‐free media containing 10 µg/mL cycloheximide for 30 min. Cell aliquots were removed at hourly intervals over the course of 6 h and stained for cell surface CCR4 expression, as above. To avoid variability in cell staining, all Ab dilutions were prepared at the same time and cells were fixed after staining using 1× CellFIX (BD CellFIX, BD Biosciences, Oxford, UK). Ligand‐induced CCR4 internalization was initially measured by incubating cells with chemokine concentrations ranging from 0 to 200 nM diluted in serum‐free media for 30 min at 37°C. A time‐course of ligand‐induced CCR4 endocytosis was carried out by stimulating cells with 100 nM chemokine and removing cell aliquots over the course of 30 min for cell surface CCR4 staining.

### 2.7 CCR4 cell surface replenishment

CCR4 internalization was induced by the stimulation of CHO‐CCR4 cells and human CD4^+^ Th2 cells with 100 nM CCL17 or 100 nM CCL22 for 30 min at 37°C. Bound chemokine was removed by washing cells in 0.5 M NaCl. Cells were then resuspended in simple media and re‐incubated at 37°C. Cell aliquots were removed at the indicated time‐points and cell surface CCR4 expression was assessed by flow cytometry.

### 2.8 Chemotaxis

Chemotaxis was assessed as previously described 21 using ChemoTx plates (Neuroprobe, Rockville MD, USA). Briefly, 0.1% BSA in RPMI 1640 was used to generate several chemokine concentrations, which were pipetted into previously blocked wells of the plate. The membrane of the apparatus was subsequently placed on top, and 20 µl of a cell suspension containing 2 × 10^5^ cells were added on top of the membrane above each well. After a 5‐h incubation at 37°C and 5% CO_2_, the membrane was removed and cell migration into the well assessed by staining with the live cell dye CellTiter Glo (Promega, Southampton, UK). Data are reported as Chemotactic Index, which is defined as the ratio of chemotactic responses to chemokine to those of media alone.

### 2.9 Statistical analysis

Data are presented as the mean ± sem of at least 3 independent experiments. All statistical tests were performed using GraphPad Prism software V6 (San Diego, CA) with the appropriate test detailed in the fig. legend. ^*^Indicates *P* ≤ 0.05, ^**^ *P *≤ 0.01, ^***^ *P *≤ 0.001, ^****^ *P* ≤ 0.0001.

## 3 RESULTS

### 3.1 Cell surface CCR4 and total CCR4 can be detected by the 10E4 Ab with *N*‐linked glycosylation of CCR4 apparent in transfectant systems

**Figure 1:**
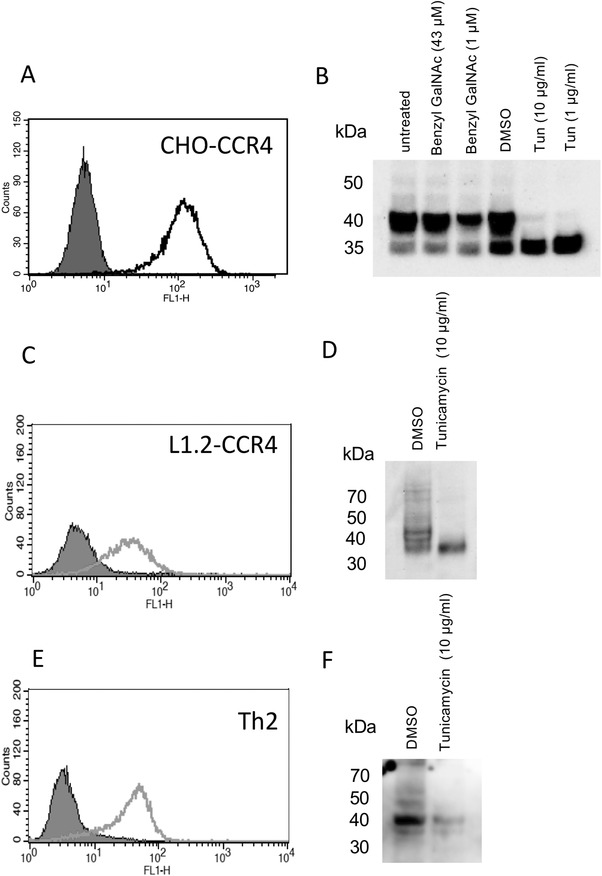
**Analysis of CCR4 expression and glycosylation on CHO‐CCR4 cells, L1.2‐CCR4 cells, and human Th2 cells**. Panels (**A**), (**C**), and (**E**) show representative histograms showing expression of CCR4 surface levels on CHO‐CCR4 cells (A), L1.2‐CCR4 cells (**C**), and human Th2 cells (**E**) as revealed by the 10E4 mAb. Isotype control staining is shown by the filled histogram. Panels (**B**), (**D**), and (**F**) show the detection of CCR4 in CHO‐CCR4 cell lysates (**B**), L1.2‐CCR4 cell lysates (**D**), and human Th2 cell lysates (**F**) as revealed by the 10E4 mAb. The effects of inhibitors of *N‐*linked and *O‐*linked glycosylation on the apparent molecular weight of CCR4 are shown. Data are representative of at least 3 independent experiments

Initially, we validated expression of CCR4 on a CHO‐K1 derived cell line engineered to express high levels of CCR4 on the cell surface.^14^ As anticipated, the anti‐human CCR4 mAb 10E4^17^, ^21^ was readily able to detect CCR4 on the cell surface (Fig. 1A). The same mAb was also able to detect CCR4 in CHO‐CCR4 cell lysates, with a major band running at around 41 kDa and a minor band running at an apparently lower molecular weight (Fig. 1B). We hypothesized that the lower molecular weight CCR4 species might reflect a non‐glycosylated form of the receptor. To test this hypothesis, we treated CHO‐CCR4 cells with inhibitors of *N*‐linked and *O*‐linked glycosylation, namely tunicamycin and benzyl GalNAc. While benzyl GalNAc had no observable effect on the apparent molecular weight of CCR4, tunicamycin treatment was seen to result in increased detection of the lower molecular weight species, concomitant with the loss of the 41 kDa band. Thus, we conclude that CCR4 undergoes *N*‐linked glycosylation in CHO cells.

To strengthen these findings, we also assessed CCR4 expression in L1.2 transfectants transiently expressing CCR4. 10E4 was able to detect CCR4 at the cell surface, albeit at lower levels than in the stable CHO‐CCR4 cell line (Fig. 1C). Tunicamycin was seen to reduce the molecular weight of the receptor, again suggestive of *N‐*linked glycosylation (Fig. 1D). 10E4 was also able to detect CCR4 on the surface of human Th2 cells (Fig. 1E) and in cell lysates from Th2 cells by Western blot (Fig. 1F). Notably, although the band ran at the expected molecular weight, it appeared much less diffuse than in the transfectant systems. Moreover, the apparent molecular weight of CCR4 was unchanged by tunicamycin treatment, suggesting that *N‐*linked glycosylation of CCR4 is restricted to expression in transfectant systems. Alternatively, given the much lower levels of CCR4 expression in Th2 cells, the 10E4 Ab may be insufficiently sensitive to detect changes in CCR4 via Western blotting.

**Figure 2:**
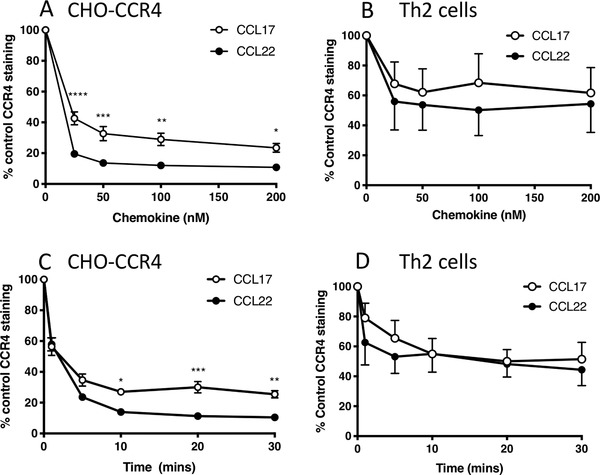
**CCL17 and CCL22 induce CCR4 endocytosis on CHO‐CCR4 transfectants and human Th2 cells in a concentration and time‐dependant manner**. Panels (A) and (B) show cell surface CCR4 expression in CHO‐CCR4 cells (**A**) and Th2 cells (**B**) following stimulation with 0–200 nM CCL17 or 0–200 nM CCL22 for 30 min at 37°C. Panels (**C**) and (**D**) show cell surface CCR4 expression in CHO‐CCR4 cells (**C**) and Th2 cells (**D**) following stimulation with 100 nM CCL17 or CCL22 for 0–30 min. Data represent the mean ± SEM of 3 independent experiments (CHO‐CCR4 cells) or 4 independent experiments (Th2 cells). Analysis where shown is by 2‐way analysis of variance (ANOVA) with Bonferroni's multiple comparisons. Significant differences between stimulation with CCL17 and CCL22 are shown

A key pathway by which GPCR responses are regulated is by means of receptor endocytosis.^3^ We therefore set out to characterize the downregulation of CCR4 in response to its endogenous ligands, CCL17 and CCL22. We used the 10E4 mAb to probe downregulation of CCR4 cell surface levels in response to CCL17 and CCL22 on the CHO‐CCR4 cell line (Fig. 2). When endocytosis of CCR4 on CHO‐CCR4 cells was examined, CCL22 was found to be the more potent chemokine with maximal CCR4 internalization occurring in response to 50 nM CCL22 compared with 200 nM CCL17 (Fig. 2A). Additionally, CCL22 was the most efficacious chemokine of the pair and induced significantly greater levels of CCR4 endocytosis compared with CCL17 at all chemokine concentrations tested. By time course experiments, internalization of CCR4 in CHO cells was found to be rapid, with the majority of receptor down‐regulated following 10 min of stimulation (Fig. 2C). Differences in the efficacy of the 2 chemokines to induce CCR4 internalization became apparent after 10 min of stimulation with 100 nM chemokine, with CCL22 again inducing significantly greater loss of cell surface CCR4 than CCL17.

The dose response and time course experiments were subsequently repeated using human Th2 cells (Figs. 2B and D). Both chemokines induced significant CCR4 internalization in Th2 cells at all concentrations tested when compared to untreated cells. Whilst there was a trend for greater internalization of CCR4 in response to CCL22 when compared with CCL17, unlike data generated with the CHO‐CCR4 line, there were no significant differences between the 2 chemokines (Fig. 2B). Similarly, both chemokines induced significant CCR4 internalization over a 30‐min time course when compared to untreated Th2 cells, and although CCL22 trended toward inducing a greater internalization of CCR4 than CCL17 at the 1 and 5 min time points; again there were no differences between the 2 chemokines (Fig. 2D).

### 3.2 CCR4 is replenished at the cell surface following ligand‐induced internalization by de novo protein synthesis

**Figure 3:**
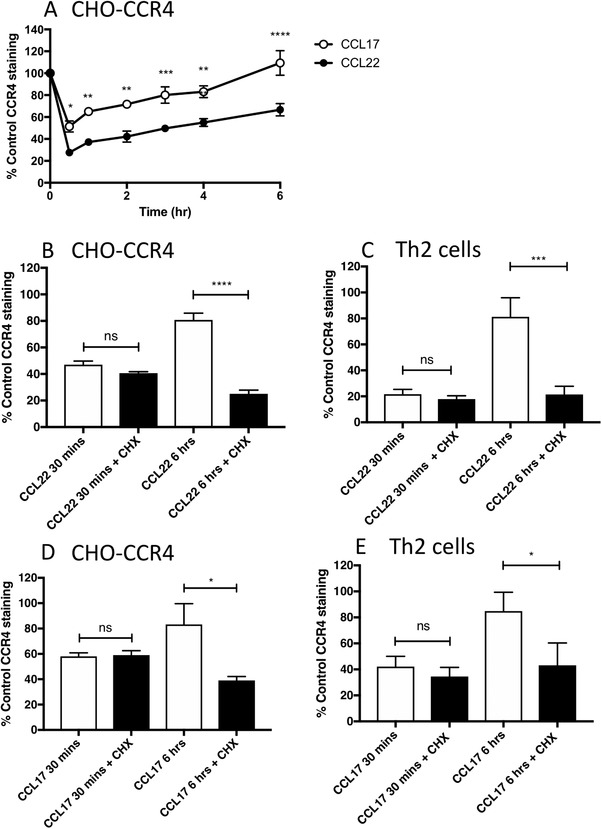
**CCR4 is replenished at the cell surface by *de novo* protein synthesis following ligand‐induced internalization**. (**A**) Cell surface CCR4 replenishment in CHO‐CCR4 cells following stimulation with 100 nM CCL17 or 100 nM CCL22 for 30 min at 37°C. Data are presented as the mean ± sem of 4 independent experiments. Panels (**B**)–(**E**) show replenishment of CCR4 at the cell surface of CHO‐CCR4 cells (**B** and **D**) and Th2 cells (**C** and **E**) following receptor internalization induced by treatment with 100 nM CCL22 (**B** and **C**) or 100 nM CCL17 (**D** and **E**). Cells were incubated in media with or without 10 µg/ml cycloheximide (CHX) during the 6‐h replenishment period. Data are presented as the mean ± sem of 3 independent experiments and was analyzed by one‐way ANOVA with Bonferroni's multiple comparisons

Following ligand‐induced internalization, cell surface chemokine receptor expression is replenished by 2 possible routes: receptor recycling and de novo protein synthesis. To distinguish between these 2 possibilities, we first investigated the dynamics of CCR4 recovery at the cell surface following internalization. CHO‐CCR4 cells were stimulated with 100 nM CCL17 or 100 nM CCL22 for 30 min to induce receptor internalization, after which chemokine was removed by washing, and cells were resuspended in media without chemokine. Receptor replenishment at the cell surface was monitored by flow cytometry. CCL22 was observed to induce significantly greater levels of CCR4 internalization than CCL17, with CCR4 returning to the cell surface very slowly, over a matter of hours rather than minutes (Fig. 3A). Receptor replenishment following treatment with either chemokine was at comparable rates and in the case of cells stimulated with CCL17, returned to the levels observed on untreated cells after 6 h. However, at the same time point, around 40% less CCR4 was present on the cell surface of CCL22‐stimulated cells.

Given the extremely slow rate of CCR4 replenishment to the cell surface, we hypothesized that CCR4 may be replenished by de novo protein synthesis rather than receptor recycling. To test this hypothesis, CHO‐CCR4 cells were re‐incubated in simple media containing 10 µg/ml cycloheximide for the duration of the replenishment period. The addition of cycloheximide had a marked inhibitory effect on receptor replenishment at the cell surface 6 h after the removal of both CCL22 and CCL17 (Fig. 3B and D). These results were also reproduced in human Th2 cells (Fig. 3C and E) suggesting that CCR4 cell surface replenishment is dependent on de novo protein synthesis.

### 3.3 CCR4 undergoes constitutive internalization in Hut78 and CHO‐CCR4 cells

**Figure 4:**
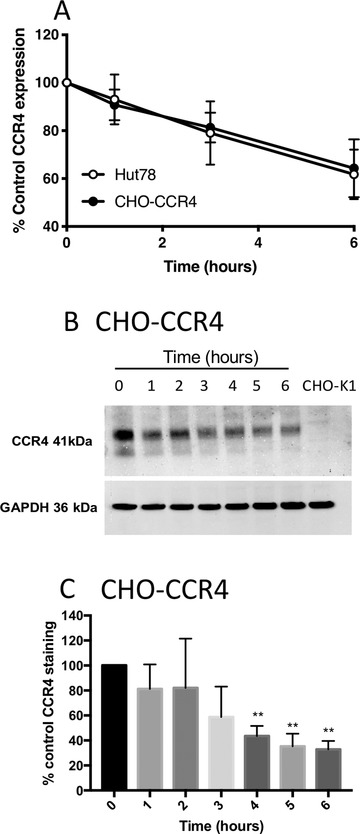
**CCR4 undergoes constitutive internalization in Hut78 and CHO‐CCR4 cells**. (**A**) Constitutive CCR4 loss from the surface of Hut78 and CHO‐CCR4 cells over a 6‐h time course. (**B**) CCR4 degradation over the same period in whole cell lysates generated from CHO‐CCR4 cells incubated in simple media in the absence of chemokine. (**C**) Densitometry analysis of the data shown in (**B**). Data are presented as the mean ± sem of 5 independent experiments that were analyzed by one‐way repeated measures ANOVA (**A**) and 2‐way repeated measures ANOVA (**C**) with Bonferroni's multiple comparisons

Constitutive receptor internalization has been reported for several chemokine receptors notably CXCR3 which is associated with Th1 inflammation.^20^ To investigate this phenomenon in the context of CCR4, Hut78 cells and CHO‐CCR4 cells were incubated in media containing 10 µg/ml cycloheximide for up to 6 h. Over the course of 6 h in the absence of exogenous additional ligand, both cell lines exhibited a loss of around 40% CCR4 cell surface staining, indicative of constitutive receptor internalization (Fig. 4A). Western blotting of samples taken at discrete time points showed that CCR4 was degraded constitutively over the course of 6 h in the absence of ligand, with a half‐life between 3 and 4 h (Fig. 4B and C).

### 3.4 Truncation of the C‐terminus of CCR4 does not affect cell surface receptor expression but significantly impairs receptor endocytosis and enhances chemotaxis

**Figure 5:**
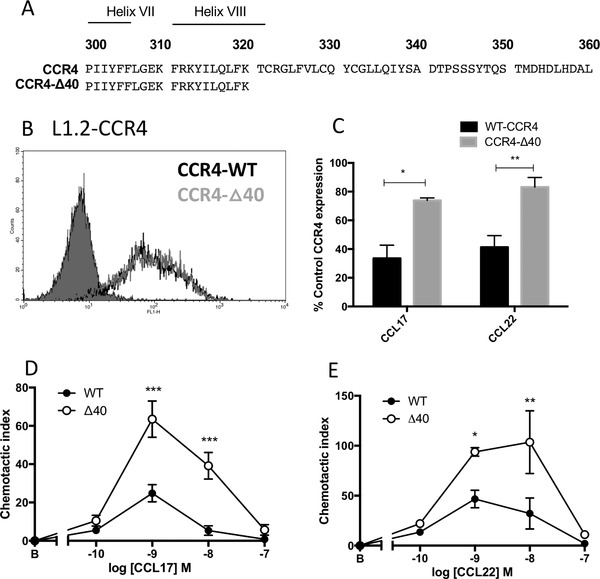
**Truncation of the CCR4 C‐terminus significantly impairs ligand‐induced receptor endocytosis with consequences for CCR4 signaling**. (**A**) A cartoon showing the C‐termini of WT CCR4 and the CCR4‐Δ40 construct. The putative positions of Helix VII and Helix VIII are also shown. (**B**) Representative histograms of cell surface anti‐CCR4 10E4 staining in L1.2 cells transiently transfected with either WT CCR4 (solid black line) or CCR4‐Δ40 (solid gray line) compared to isotype control‐stained cells (filled histogram). (**C**) The percentage of cell surface receptor expression in L1.2 transfectants expressing WT CCR4 or CCR4‐Δ40 following incubation with 100 nM CCL17 or 100 nM CCL22 for 30 min at 37°C. (**D**) and (**E**) Chemotaxis of L1.2 transfectants expressing WT CCR4 or CCR4‐Δ40 toward increasing concentrations of CCL17 (**D**) and CCL22 (**E**). Data are presented as the mean ± sem of 3 experiments, which were analyzed by 2‐way repeated measures ANOVA with Bonferroni's multiple comparisons

The intracellular C‐terminal region of chemokine receptors is a key region involved in regulating receptor turnover, by virtue of numerous phosphorylation sites which are the target of G protein‐coupled receptor kinases (GRKs).^22^ To examine the role of this motif in the regulation of CCR4 expression, site‐directed mutagenesis was undertaken to generate a CCR4 truncation mutant which we named CCR4‐Δ40. In this mutant, truncated at Lysine 320 by the introduction of a premature stop codon, all potential phosphorylation sites (serine/threonine residues) within the 40‐most C‐terminal residues were removed (Fig. 5A).

Plasmids encoding either WT CCR4 or CCR4‐Δ40 were transiently transfected into L1.2 cells. As shown in Fig. 5B, transfectants expressed both constructs at identical levels on the cell surface, as determined by flow cytometry. The 2 receptors responded differently, however, in receptor endocytosis assays. WT CCR4 underwent significant endocytosis in response to stimulation with both 100 nM CCL17 and 100 nM CCL22 with a 60% reduction in cell surface levels (Fig. 5C). In contrast, no significant endocytosis of the CCR4‐Δ40 construct was observed in response to treatment with either chemokine for 30 min (Fig. 5C). C‐terminal CCR4 truncation also had consequences for the chemotactic responses of L1.2 transfectants. Cells expressing the CCR4‐Δ40 construct had significantly more efficacious chemotactic responses toward both CCL17 (Fig. 5D) and CCL22 (Fig. 5E). Specifically, C‐terminal CCR4 truncation enhanced L1.2 chemotaxis toward the optimal concentrations of CCL17 and CCL22 by 2‐ to 3‐fold (Figs. 5D & E), although the responses were still bell‐shaped.

### 3.5 C‐terminal CCR4 truncation has no effect on constitutive degradation

**Figure 6:**
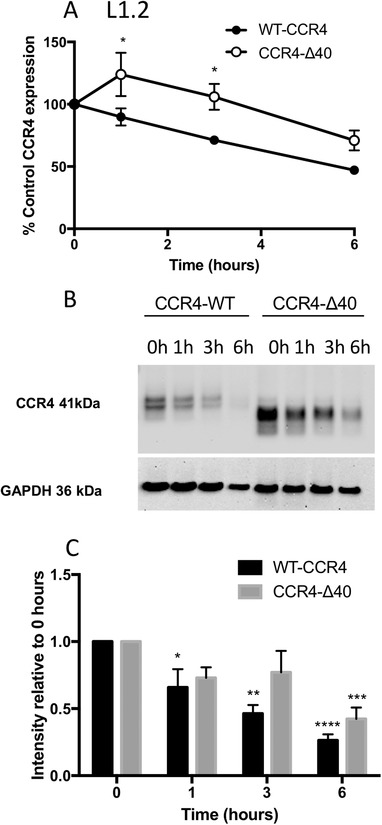
**Constitutive degradation of CCR4 is unimpaired by C‐terminal truncation**. (**A**) Constitutive internalization of CCR4 in L1.2 transfectants over a 6‐h period. Data shown are the mean ± sem of 7 independent experiments. (**B**) Western blot showing CCR4 degradation over the same 6‐h period in whole cell lysates from L1.2 transfectants expressing WT CCR4 and CCR4‐Δ40. The blot is representative of 5 independent experiments. (**C**) Densitometry analysis of receptor degradation. Data were analyzed by 2‐way ANOVA with Bonferroni's multiple comparisons

Given the differences in the internalization responses of wild‐type and truncated CCR4, it was of interest to determine whether C‐terminal truncation also affects other processes that may influence CCR4 turnover, namely constitutive endocytosis and degradation. L1.2 transfectants expressing both WT CCR4 and the CCR4‐Δ40 construct were treated with cycloheximide and CCR4 expression was examined by flow cytometry and Western blotting at distinct time points. Cell surface expression of both constructs in L1.2 cells decreased over the incubation period at similar rates as judged by the parallel traces (Fig. 6A). Notably, cell surface expression of the CCR4‐Δ40 construct increased after 1 h incubation in the presence of cycloheximide before decreasing to 80% of starting levels over the course of 6 h. In comparison, levels of WT CCR4 decreased to 60% of control cells after 6 h. This was mirrored by Western blot analysis, where much higher basal levels of CCR4 expression were observed in the lysates of CCR4‐Δ40 transfectants compared to WT CCR4 transfectants (Fig. 6B) despite lysates being made from identical numbers of cells, which expressed similar cell surface levels of receptor (Fig. 5B). When CCR4 protein was examined over the 6 h time course both constructs exhibited similar patterns of CCR4 degradation over time (Fig. 6B) with the rate of receptor degradation over time directly comparable for both constructs (Fig. 6C). This suggests that the 40‐most C‐terminal residues of CCR4 do not play a major role in the constitutive endocytosis or degradation of the receptor.

**Figure 7:**
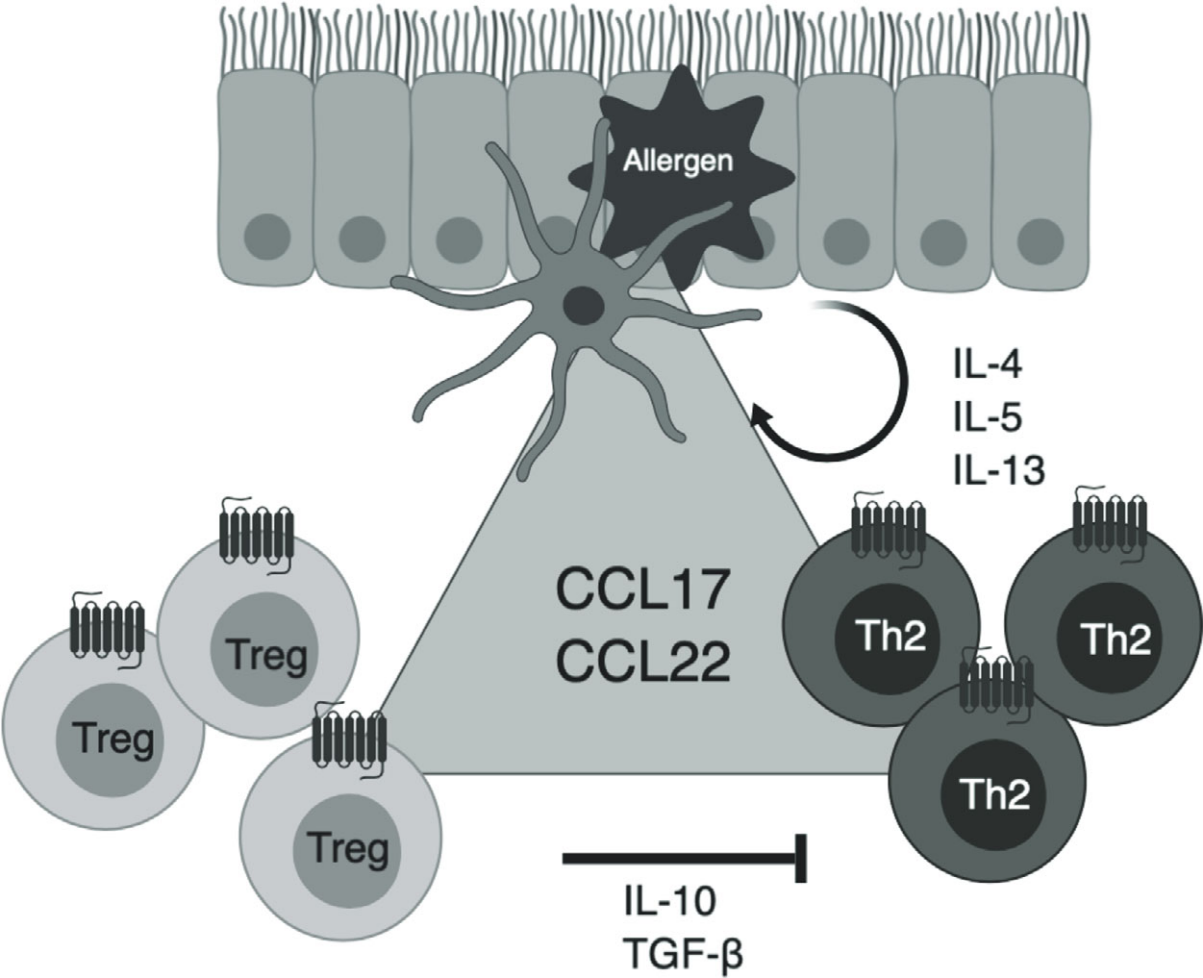
**Schematic outlining the role of CCR4 in allergic inflammation**. Allergen challenge leads to the activation of dendritic cells in the airway lumen which secrete the CCR4 ligands CCL17 and CCL22. The chemokines serve to recruit CCR4^+^ Th2 cells that secrete the pro‐inflammatory cytokines IL‐4, IL‐5, and IL‐13. These cytokines act on eosinophils, airway smooth muscle cells and mucus‐producing goblet cells in the airways and result in many of the hallmark features of allergic inflammation. IL‐4 also induces the expression of CCL17 and CCL22, thereby perpetuating Th2 cell recruitment by a positive feedback loop. Both chemokines also recruit CCR4^+^ Treg cells that produce IL‐10 and transforming growth factor (TGF)‐β. These 2 cytokines act to dampen down Th2‐induced inflammation. Loss of Treg functions leads to dysregulated Th2 activation, uncontrolled inflammatory responses and the development of autoimmunity. Fine‐control of CCR4 signaling by manipulation of cell‐surface levels is therefore necessary to effectively maintain homeostasis and control inflammation.

## 4 DISCUSSION

Despite the licensing of the CCR5 antagonist maraviroc and the CXCR4 antagonist plerixafor for the inhibition of HIV‐1 infection 23 and stem cell mobilization respectively,^24^ no chemokine receptor antagonists to date have shown efficacy for the treatment of inflammatory conditions. This is despite intense efforts by many groups worldwide. Many different viewpoints have been put forward as to why this might be, with one of the most compelling reasons being an incomplete understanding of the biology of the target and its precise role in human disease.^25^ CCR4 is a case in point.

CCR4 is expressed by Th2 cells 5, 6, 7 with the number of CCR4^+^ T cells reported to be significantly upregulated in the peripheral blood and airways of patients with severe and moderate asthma, despite ongoing corticosteroid treatment.^26^ Combined with a substantial body of evidence detailing the production of both CCL17 and CCL22 in the allergic lung (reviewed by Solari and Pease 27), it is apparent that targeting CCR4 in allergic disease is attractive. The only small molecule CCR4 antagonist to have been tested in humans was the compound GSK2239633,^28^ which although well tolerated, suffered from poor oral bioavailability and managed only 74% receptor occupancy, substantially below the continual 90% occupancy of all receptors predicted to be needed for effective treatment of inflammation.^29^ Moreover, the expression of CCR4 on regulatory T cells (Tregs) suggests that even if potent, high occupancy CCR4 antagonists were to be developed, then global CCR4 blockade may be counterproductive as the natural immunosuppressive nature of CCR4^+^ Tregs would also be impeded.^30^, ^31^ This is evidenced by the severe autoimmune reactions such as Stevens‐Johnson syndrome reported in adult T cell leukemia/lymphoma (ATLL) patients treated with the anti‐CCR4 mAb, mogamulizumab.^32^, ^33^ This treatment results in the loss of all CCR4^+^ cells and highlights the biological requirements for fine control of CCR4 signaling during both homeostasis and disease. Understanding just how CCR4 signaling is regulated may provide opportunities for more precise targeting of this receptor.

In this study, we have shown that CCR4 was endocytosed following exposure to both CCL17 and CCL22 with CCL22 appearing to be the most efficacious ligand when assays were performed in CHO‐CCR4 cells. This was not the case in human Th2 cells where although both ligands showed efficacy, there were no significant differences in their ability to downregulate CCR4. This could be an apparent example of tissue bias,^34^ although it should be noted that cell surface expression levels of CCR4 on primary human Th2 cells were much lower than in the stable CHO‐CCR4 transfectant cell line. As such, the window of CCR4 expression for the internalization assay was reduced for these samples. Coupled with greater variability in CCR4 expression levels on human Th2 cells, the significance of this observation remains to be determined.

We have also shown that CCR4 cell surface levels are strictly regulated. CCR4 is endocytosed in the presence or absence of ligand and once internalized, it is targeted for degradation, being slowly replenished at the cell surface by de novo protein synthesis rather than undergoing receptor recycling. This is in contrast to an earlier study by Mariani and colleagues that found cycloheximide to have no effect on CCR4 cell surface replenishment and concluded that CCR4 is recycled following internalization by ligand.^13^ These findings appear to be incompatible with the extremely slow rate of cycloheximide‐sensitive replenishment we observed here, using both CCR4 transfected cells and Th2 cells. It is notable that although the authors of the earlier study used a 5‐fold higher concentration of cycloheximide that was shown to be effective here, their study lacked a positive control for the efficacy of cycloheximide. Thus, 1 potential explanation for the discrepancies is that the cycloheximide used in the earlier study was ineffective, or inadvertently used at a lower concentration than that reported. Other differences in the 2 protocols used include the source of recombinant chemokines employed (Peprotech EC vs Dictagene) and the protocol used for Th2 generation. One previously reported difference in the studies is the Ab used to determine CCR4 expression. We have previously shown that the 10E4 Ab used in our studies here recognizes a conformationally dependent epitope of CCR4 that can interact with both CCL17 and CCL22 21 and varies from the 1G1 Ab used by Mariani et al.^13^

The detection of both non‐glycosylated and *N‐*linked glycosylation forms of CCR4 by Western blot is intriguing. CCR4 was shown in the CHO cell and L1.2 cell backgrounds to undergo *N*‐linked glycosylation, as indicated by the broad band observed on SDS‐PAGE and the sensitivity to culture with tunicamycin. However, we were unable to extend these observations to human Th2 cells, with tunicamycin having no discernible effect on the apparent molecular weight of CCR4. Given that *N‐*linked glycosylation occurs within the Golgi,^35^ an open question is where the non‐glycosylated form of CCR4 resides within the cell and whether it has a function. Several other chemokine receptors are known to undergo glycosylation, including CXCR4,^36^ CXCR2,^37^ and CCR7^38^). In the case of CXCR4, *N*‐linked glycosylation within the N‐terminus and ECL2 is required for efficient binding of ligand to CXCR4.^36^ At CCR7, different glycosylation patterns of the receptor affected receptor endocytosis and signaling.^38^ For example, de‐glycosylated CCR7 mutants were less susceptible to endocytosis but more sensitive to chemokine‐mediated chemotaxis.^38^ Glycosidase secretion by dendritic cells was also shown to de‐glycosylate CCR7 on T cells, resulting in enhanced chemotaxis.^38^ Similarly, glycosylation may be another means by which CCR4 expression and function is modulated.

We also observed that the C‐terminus of CCR4 was required for the regulation of CCR4 turnover in response to ligand. L1.2 transfectants lacking the final 40 amino acids of the receptor C‐terminus had impaired internalization in response to either CCL17 or CCL22, resulting in enhanced chemotaxis toward CCL17 and CCL22. In contrast to ligand‐induced internalization, the constitutive endocytosis of CCR4 in L1.2 cells was unaffected by C‐terminal truncation, suggesting that the 2 processes are affected by different mechanisms. Although we did not rule out the potential for autologous cell production of CCL17 or CCL22 to drive constitutive endocytosis, this would appear to be unlikely given the identical patterns of endocytosis seen in 3 quite different cell lines, the human T cell line Hut78 and the 2 non‐human transfectant systems L1.2 and CHO.

Curiously, a consistent finding was the apparent increase in cell surface levels of the Δ40 CCR4 construct at the 1‐h time point following cycloheximide treatment (Fig. 6A). This was not supported when total Δ40 protein expression was examined by Western blot (Fig. 6B) suggesting that the observation may reflect a delay in trafficking of nascent Δ40 CCR4 to the surface, perhaps by reduced interaction with an unknown chaperone. At the 0‐h time‐point, we hypothesize that the surface of the L1.2 transfectants is saturated with receptor, explaining why cell surface expression levels of CCR4‐WT and CCR4‐Δ40 transfectants are comparable (Fig. 5B). The increase in the cell surface expression of the CCR4‐Δ40 transfectant with 1‐h of cycloheximide treatment (Fig. 6A) may reflect a delay in the export of accumulated intracellular receptor to the cell surface as space becomes available at the cell surface as a result of constitutive receptor internalization. The portion of the C‐terminus removed by truncation contains 5 serine and 4 threonine residues, all of which have the potential to be phosphorylated by GRKs following engagement with ligand. C‐terminal phosphorylation has previously been shown to be key for the endocytosis of several chemokine receptors, notably CXCR4. In WHIM (warts, hypogammaglobulinemia, infections, and myelokathexis) syndrome, heterozygous mutations in the C‐terminus of CXCR4 result in a similar truncation to the one we describe here, which ablates the binding of GRK3 and GRK6, leading to defective CXCR4 phosphorylation and internalization and enhanced chemotactic responses to ligand.^39^

The effects of truncation on ligand‐induced endocytosis also closely mirror the effects of CCR4‐truncating mutations observed in a study of ATLL in which ligand‐induced CCR4 internalization in ATLL cell lines was impaired by C‐terminal truncation at glutamine 330 (CCR4‐Q330), with a concomitantly enhanced chemotactic response toward CCL17 and CCL22. Importantly, the gain‐of‐function CCR4 phenotype in ATLL cells gave the cells a competitive advantage over cells expressing wild‐type CCR4, leading to increased growth and survival.^40^ The parallels between CCR4‐Q330 and CCR4‐Δ40 could perhaps be predicted given the close proximity of the 2 truncations at positions 320 (CCR4Δ40) and 330 (CCR4‐Q330). What is remarkable, however, is that both truncation mutants are still able to signal via G‐proteins to drive chemotaxis. We have previously shown that mutation of helix VIII within the C‐terminus of CCR4 at lysine 310 (K310N) completely ablated chemotaxis in response to CCL17, while robust chemotaxis was observed in response to CCL22, albeit with reduced potency.^21^ These results suggest that, in conjunction with the conserved DRYLAIV motif within ECL2, the residues within helix VIII (Fig. 5) are also likely to be involved in G‐protein coupling and signaling, with an absolute requirement for residue K310 for activation of CCR4 by CCL17.

Why might CCR4 expression be under such strict levels of control? The regulation of CCR4 we observed is reminiscent of that of the Th1‐associated receptor CXCR3,^41^, ^42^ which our group has previously shown undergoes constitutive endocytosis, degradation, and replenishment at the cell surface by de novo synthesis.^20^ Th2 cells are a notable source of IL‐4 and IL‐13, which induce the expression of CCL17 and CCL22 by monocytes and Mϕs, allowing for a positive‐feedback loop in both Th2 and Treg recruitment (Fig. 7), as previously put forward by Bonecchi et al.^41^ As such, it may be important to conclusively "switch off" CCR4 signaling to effectively maintain both homeostasis and the control of inflammatory responses by Tregs.

In summary, we have shown that CCR4 expression is subject to multiple levels of regulation, explained by a need to prevent inappropriate activation of a key chemokine receptor that is expressed by both Th2 and Treg cells. CCR4 expression and signaling was tightly regulated by the biased, endogenous ligands CCL17 and CCL22, which induced receptor internalization and degradation, followed by slow cell surface receptor replenishment. Finally, we identified the final 40 amino acids of the C‐terminus as a key region involved in receptor endocytosis and intracellular receptor trafficking and signaling. The specific targeting of this region of CCR4 is plausible by the use of previously identified small molecules that recognize this region.^43^ It will be interesting to see if such compounds interfere with regulation of CCR4 as might be predicted from this study.

## AUTHORSHIP

C.A.A., P.P., R.S., and J.E.P. contributed to the conception and design of the study. C.A.A., P.P., J.M.V., and R.M.P. performed the experiments. C.A.A., J.M.V., and J.E.P. conducted data analysis and interpretation of results. C.A.A., R.S., and J.E.P. wrote the manuscript.

## DISCLOSURE

The authors declare no conflict of interest related to the content of this work.
